# Selenium Catalyzed Oxidation of Aldehydes: Green Synthesis of Carboxylic Acids and Esters

**DOI:** 10.3390/molecules200610496

**Published:** 2015-06-08

**Authors:** Luca Sancineto, Caterina Tidei, Luana Bagnoli, Francesca Marini, Eder J. Lenardão, Claudio Santi

**Affiliations:** 1 Group of Catalysis and Organic Green Chemistry, Department of Pharmaceutical Sciences, University of Perugia, Via del Liceo 1, Perugia 06100, Italy; E-Mails: sancineto.luca@gmail.com (L.S.); caterinatidei@libero.it (C.T.); luana.bagnoli@unipg.it (L.B.); francesca.marini@unipg.it (F.M.); 2 Laboratório de Síntese Orgânica Limpa (LASOL), Centro de Ciências Químicas, Farmacêuticas e de Alimentos (CCQFA), Universidade Federal de Pelotas (UFPel), P.O. Box 354, Pelotas 96010-900, Brazil; E-Mail: lenardao@ufpel.edu.br

**Keywords:** aldehydes, oxidation, selenium, catalysis, carboxylic acids, esters, hydrogen peroxide, green chemistry

## Abstract

The stoichiometric use of hydrogen peroxide in the presence of a selenium-containing catalyst in water is here reported as a new ecofriendly protocol for the synthesis of variously functionalized carboxylic acids and esters. The method affords the desired products in good to excellent yields under very mild conditions starting directly from commercially available aldehydes. Using benzaldehyde as a prototype the gram scale synthesis of benzoic acid is described, in which the aqueous medium and the catalyst could be recycled at last five times while achieving an 87% overall yield.

## 1. Introduction

Oxidation reactions play an important role in modern organic chemistry and during the recent decades, several methods have been developed in response to an increasing demand for selective mild and eco-friendly procedures [1,2]. The carboxylic acid moiety is a common functional group in a number of organic molecules, including drugs, fine chemicals and industrially interesting compounds. It is usually synthesized through oxidation reactions starting from reduced precursors such as alcohols [3], aldehydes [4] or ketones [5]. Among these procedures, the transformation of aldehydes into carboxylic acids is a very useful chemical reaction and many successful methods have been developed for that purpose. For a long time, toxic heavy metals and hazardous compounds such as potassium permanganate, chromates and chlorite have been used in stoichiometric amounts and organic solvents were usually required. More sustainable and atom efficient procedures have been adopted, especially concerning the use of hydrogen peroxide as greener oxidant, which however must be employed in the presence of organic [6], inorganic [7] or transition metal-based catalysts [8,9,10,11,12] in order to allow the oxygen-transfer reactions to proceed, usually in organic solvents. 

During the last ten years, we have investigated in depth the possibility of using water as a reaction medium for organoselenium and organosulfur chemistry [13,14,15,16,17,18,19,20,21,22,23,24,25] focusing a part of our efforts on the use of organoselenenic acids as catalysts for new bio-inspired and eco-friendly oxidation reactions [17,21,24]. As in the glutathione peroxidase catalytic cycle, selenium reduces hydrogen peroxide promoting the oxidation of functionalized organic substrates. This general protocol has been successfully applied to the oxidation of thiols into the corresponding disulfides and olefins into the corresponding vicinal diols [17,21,24]. In all the cases the reaction was efficiently effected in water suspensions that generally resulted to be a convenient reaction medium enhancing the efficiency of the catalysts, reducing the undesired disproportionation of hydrogen peroxide and facilitating the possibility to reuse the catalyst and the medium several times without any kind of purification and/or activation [24]. Recently, other authors also reported different applications in which the selenium catalyst is involved in the oxygen transfer oxidation from hydrogen peroxide to organic substrates demonstrating a current and growing interest in the field [26,27].

In this paper, we describe a new and versatile method to achieve the oxidation of aldehydes using aqueous hydrogen peroxide in the presence of catalytic amounts of diphenyl diselenide as pre-catalyst demonstrating the possibility of reusing the aqueous medium at least five times in a gram scale preparative protocol. As an extension of the reaction, by changing the reaction medium from water to an alcohol, the direct synthesis of esters from aldehydes can be achieved.

## 2. Results and Discussion

**Scheme 1:**
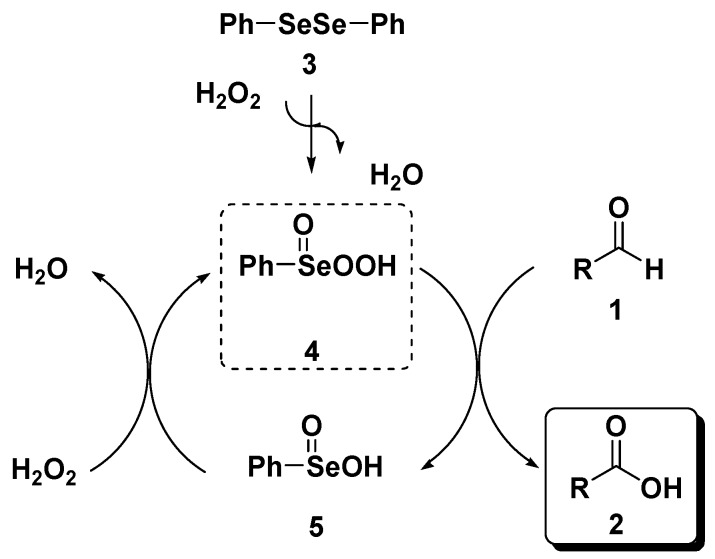
Diphenyl diselenide-mediated oxidation of aldehydes.

Preliminary investigations were carried out using, as probe reaction, the oxidation of benzaldehyde (**1a**) into the corresponding carboxylic acid **2a** using a stoichiometric amount of diluted aqueous hydrogen peroxide under various reaction conditions (summarized in Table 1). As can be seen in Table 1, benzaldehyde (**1a**) is converted into benzoic acid (**2a**) using one equivalent of 10% (or 20%) aqueous hydrogen peroxide and 1% (or 2%) of diphenyl diselenide (**3**) without any organic co-solvent (entries 2–5). Because all the reactions occurred in non-homogeneous phases, they were effected under vigorous stirring (800 rpm). Diselenide reacts with hydrogen peroxide affording the oxidized seleninic (**5**) and perseleninic acid (**4**) forms that reasonably, are the actual catalyst of the process. Its presence is pivotal in the oxidation mechanism and only trace amounts of benzoic acid were recovered when the reaction was effected without diphenyl diselenide (entry 1). The proposed catalytic cycle is depicted in Scheme 1.

**Table 1:** Oxidation of benzaldehyde (**1a**) into benzoic acid (**2a**). 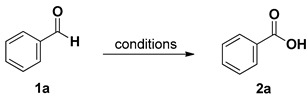

Entry	Catalyst	Oxidant	Solvent	Time (h)	Yield ^a^
1	none	10% H_2_O_2_	H_2_O	6	trace
2	1% (PhSe)_2_	10% H_2_O_2_	H_2_O	4	56%
3	1% (PhSe)_2_	10% H_2_O_2_	H_2_O	6	61%
4	1% (PhSe)_2_	20% H_2_O_2_	H_2_O	6	85%
5	2% (PhSe)_2_	10% H_2_O_2_	H_2_O	6	>99%
6 ^b^	2% (PhSe)_2_	10% H_2_O_2_	H_2_O	6	trace
7	4% SeO_2_	10% H_2_O_2_	H_2_O	6	trace
8	2.5% (PhSe)_2_	10% H_2_O_2_	THF	6	80%
9	4% HCl	10% H_2_O_2_	H_2_O	6	30%

^a^: All the reactions were carried out at room temperature; ^b^: The reaction was carried out in the presence of a stoichiometric amount of TEMPO.

The best conditions, summarized in Table 1, entry 5, afforded the quantitative formation of **2a** in six hours. The yields resulted to be sensitive to and affected by a number of parameters: (a) the reaction time (entry 2), (b) the amount of the catalyst (entry 3) and (c) the concentration of the oxidant (entry 4). The reaction did not afford the desired product in the presence of (2,2,6,6-tetramethylpiperidin-1-yl)oxyl (TEMPO) as radical trapping (entry 6), indicating a reasonable involvement of radical intermediates in the mechanism. Mlochowsky *et al.*, reported the selenium(IV) oxide-mediated oxidation of aldehydes using overstoichiometric amounts of H_2_O_2_ in refluxing THF [28]; however, we proved that under the conditions optimized for the benzenseleninic acid, only trace amounts of benzoic acid were observed (entry 7). Furthermore, this preliminary investigation also demonstrated that the aqueous medium is superior respect to the organic one (entry 8 *vs.* entry 5). When THF was used as the solvent in the presence of benzenseleninic acid (**5**) as catalyst, according to the conditions previously reported by Choi *et al.* [29] lower yields were obtained, despite the use of a higher amount of catalyst. When diphenyl diselenide was substituted by HCl (4%) a 30% yield of **2a** was obtained, evidencing only a slightly catalytic effect of the acidity in effecting the oxidation.

With the optimized conditions in hands, we investigated the scope of the reaction employing variously substituted aromatic aldehydes bearing electron withdrawing and donating groups, as well as aliphatic and conjugated ones. The results are summarized in Table 2.

**Table 2:** Oxidation of aldehydes to carboxylic acids. 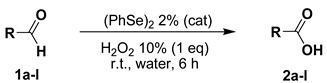

Entry	Substrate		Product		Yield (%) ^a^
1	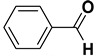	**1a**	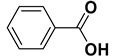	**2a**	>99
2	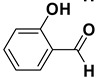	**1b**	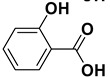	**2b**	75
3	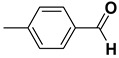	**1c**	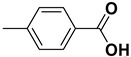	**2c**	85
4	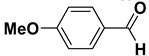	**1d**	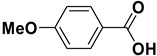	**2d**	80
5	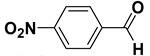	**1e**	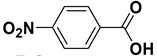	**2e**	30, 88 ^b^
6	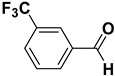	**1f**	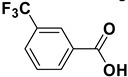	**2f**	99
7	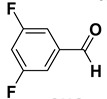	**1g**	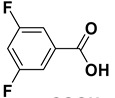	**2g**	78
8	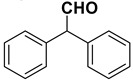	**1h**	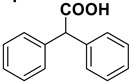	**2h**	80
9	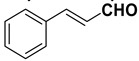	**1i**	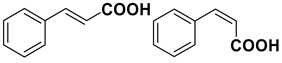	**2i**	20 (*E*), 65 (*Z*) ^c^
10	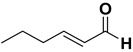	**1j**	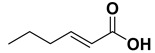	**2j**	>99
11	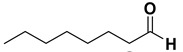	**1k**	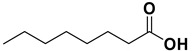	**2k**	>99 ^d^
12	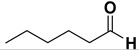	**1l**	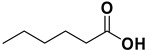	**2l**	>99 ^d^

^a^: Yields of isolated products; ^b^: The reaction was carried out for 24 h; ^c^: E/Z ratio = 24:76 by ^1^H-NMR of the crude; ^d^: The reaction was carried out for 3 h.

The proposed protocol showed a broad applicability, as both aromatic (compounds **1a**–**g**) and aliphatic aldehydes (compounds **1h**, **l**) were efficiently converted into the corresponding carboxylic acids **2a**–**g** and **2h**, **l**, respectively, in yields ranging from good to excellent. With the only exception of *p*-nitrobenzaldehyde **1e** (Table 2, entry 5), that required 24 h of reaction time to afford appreciable amount of **2e**, for the aromatic aldehydes the presence of the functional groups with different electronic properties did not influence, in general, the outcome of the reaction. Electron withdrawing group-containing aldehydes **1f** and **1g** yielded the target acids in 99 and 79% yields, while electron donating group-containing ones (compounds **1b**–**d**) gave the oxidized products in yields ranging from 75% to 85%. 2,2-Diphenylacetaldehyde (**1h**) was converted into the target acid **2h** in 80% yield indicating that the steric hindrance has a minor influence on the reaction. Aliphatic non-conjugated aldehydes resulted reasonably more reactive, affording the carboxylic acids **2k** and **2l** quantitatively after 3 h instead of six (Table 2, entries 10 and 11). It is interesting to observe that the oxidation of *trans*-cinnamaldehyde (**1i**) gave the target cinnamic acid **2i** in good yield but unexpectedly as a *Z*/*E* isomer mixture, in which the less stable *Z*-isomer was the major product. On the contrary, the α,β-unsaturated (*E*)-hex-2-enal (**1j**) when subjected to similar conditions, afforded, in excellent yield, only the *E*-**2j** isomer. In order to better investigate this finding, a series of experiments were carried out and the results are summarized in Table 3. The photoisomerization of *E*-cinnamic acid is the most commonly used procedure, among the few reported [30], for the preparation of its *Z* isomer. To rule out photoactivation or light contribution to the isomerization of *E*-cinnamic acid, the oxidation of cinnamaldehyde (**1i**) was performed under strict dark conditions; however, the same results were obtained (Table 3, *cf*. entries 1 and 2).

**Table 3:** Studies on the isomerization of cinnamic derivatives. 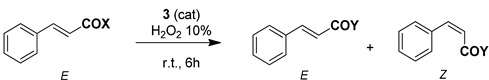

Entry	X	% of 3	H_2_O_2_	*E-*Y	*Z-*Y
1	H	2	1 eq	OH (24%)	OH (76%)
2 ^a^	H	2	1 eq	OH (27%)	OH (73%)
3	H	0	1 eq	H (100%)	
4 ^b^	H	2	none	H (100%)	
5	OH	0	1 eq	OH (100%)	
6	OH	2	1 eq	OH (100%)	
7 ^c^	H	2	1 eq	H (100%)	

^a^: The reaction was carried out under strict dark conditions; ^b^: The reaction was carried out in absence of hydrogen peroxide; ^c^: The reaction was performed in the presence of a stoichiometric amount of TEMPO.

As expected, both hydrogen peroxide and the catalyst **3** are required to obtain the oxidation/isomerization (Table 3, entries 3 and 4) and no isomerization was observed starting from *trans*-cinnamic acid (Table 3, entries 5 and 6), neither with nor without the catalyst. This indicates that it is reasonable to assume that the isomerization and the oxidation reactions share the same mechanism. Even if we do not have at this time a clear explanation for the mechanism, the involvement of radical intermediate has been proven by performing the oxidation of *trans*-cinnamaldehyde (**1i**) in the presence of the radical trap TEMPO (Table 3, entry 7), whereby only starting material was recovered after six hours, as in the case of the previously discussed example using benzaldehyde (**1a**, Table 1, entry 7). 

Recently, Pathak *et al.*, reported that depending on the substrate, aldehydes can be converted into the corresponding esters by using an over-stoichiometric amount of concentrated hydrogen peroxide (50%) in the presence of alcohols and heating at 70–75 °C for 5–8 h [31]. In order to evaluate the ability of diphenyl diselenide (**3**) to catalyze this reaction, we subjected the aldehyde **1a** to the reaction with 2% of **3** at 50 °C in the presence of a stoichiometric amount of both hydrogen peroxide (30%) and methanol. After only two hours the reaction was complete evidencing a positive effect of the catalyst on allowing the conversion to proceed under considerably milder conditions and in shorter reaction times (90% yield in 2 h at 50 °C *vs.* 75% yield in 5 h at 75 °C [29]; Table 4, entry 1).

**Table 4:** Esterification of aldehydes. 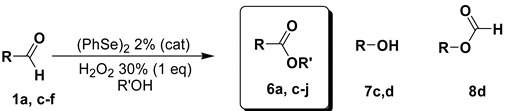

Entry	R		R′	T °C	Conv (%)	Yield (%) ^a^		
						6	7	8
1	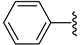	**1a**	–CH_3_	50	>99	90 (**6a**)	-	-
2	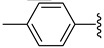	**1c**	–CH_3_	50	>99	75 (**6c**)	15 (**7c**)	-
3	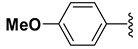	**1d**	–CH_3_	50	44	10 (**6d**)	35 (**7d**)	-
				0	97	-	15 (**7d**)	80 (**8d**)
4	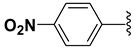	**1e**	–CH_3_	50	>99	95 (**6e**)	-	-
5	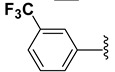	**1f**	–CH_3_	50	>99	96 (**6f**)	-	-
6	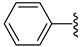	**1a**	–C_2_H_5_	50	92	87 (**6g**)	-	-
7	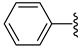	**1a**	–C_3_H_7_	50	85	80 (**6h**)	-	-
8	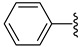	**1a**	–*i*C_3_H_7_	50	81	77 (**6i**)	-	-
9	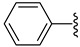	**1a**	–(CH_2_)_6_-	50	95	70 (**6j**)	-	-

^a^ Yields of isolated products.

With the optimized conditions of the esterification in hands, we explored the scope of the reaction evaluating a panel of aldehydes and different alcohols. The results are collected in Table 4. The oxidation occurred in excellent yields for benzaldehyde (**1a**) and for aldehydes bearing electron withdrawing groups (Table 4, entries 1, 4 and 5), affording the corresponding methyl esters **6a**, **6e** and **6f** (in up to 95% yields). In the cases of *p*-methylbenzaldehyde (**1c**, entry 2) and *p*-methoxybenzaldehyde (**1d**, entry 3), besides the target methyl esters **6c** and **6d**, we observed the formation of the corresponding *p*-cresol (**7c**) and *p*-methoxyphenol (**7d**), presumably as a result of a competitive Dakin-like reaction facilitated by the electron-donating character of the substituent at the *para*-position respect to the carbonyl group of the aldehyde. The nature of the competitive mechanism is confirmed by the isolation of the formyl derivative **8d** (80% yield) when the oxidation of **1d** was effected at 0 °C (Table 4, entry 3).

Beside methanol, linear, branched and cyclic aliphatic alcohols were reacted with **1a** yielding the corresponding esters **6g**–**j** in good to excellent isolated yields, showing only a slight detrimental effect of the steric hindrance in the secondary alcohols respect to the primary ones (Table 4, entries 8 and 9 *vs.* entries 1, 6 and 7).

In order to confirm the actual involvement of the *in situ*-formed benzenselenenic acid a representative number of reactions were repeated using 4 mol % of preformed **5**, observing no appreciable difference in yields and reaction times either for the synthesis of carboxylic acids or esters. In addition, the mixture resulting from the treatment of diphenyl diselenide with hydrogen peroxide in a molar ratio of 1:5 in water has been analyzed by ^77^Se-NMR using for the NMR lock and shimming procedures a sealed capillary tube containing DMSO-*d*_6_. The spectra evidenced three peaks at 1024, 1174 and 1293 ppm, respectively. The chemical shift of 1174 ppm can be unambiguously assigned to PhSeO_2_H by comparison with the spectra obtained from the commercially available original sample; beside that, it can be supposed the presence of a less oxidized specie, PhSeOH (1024 ppm) and an over-oxidized one, like PhSeO_3_H (1293 ppm) that confirm the proposed mechanism depicted in Scheme 1.

Considering the growing interest in protocols and procedures designed to minimize waste production, we investigated the recyclability of the aqueous medium containing the water-soluble catalyst **5**, evaluating the efficiency in terms of conversion and overall yield in the oxidation of **1a** for a number of five subsequent reactions affording a gram scale synthesis of **2a**.

**Figure 1:**
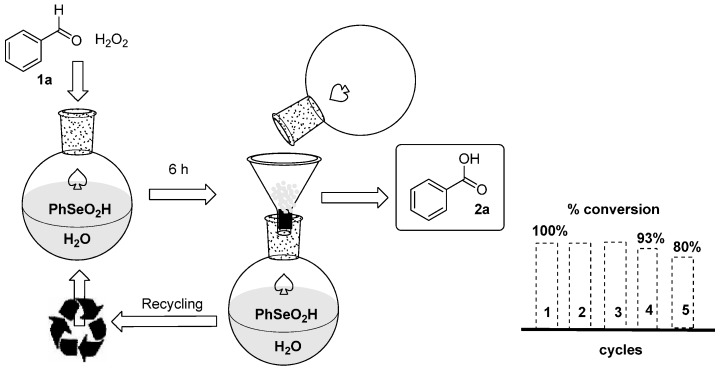
Recyclability of the aqueous medium and the catalyst in the synthesis of benzoic acid (**2a**).

**Figure 2:**
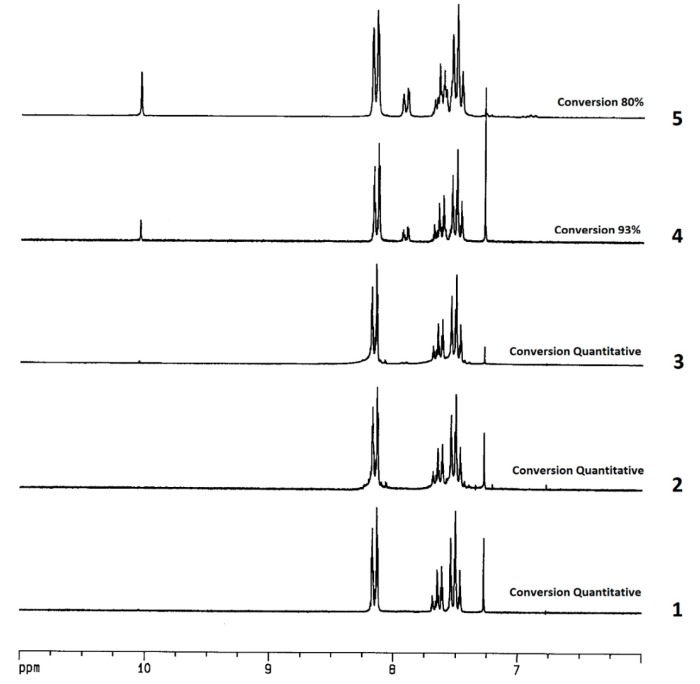
^1^H-NMR (200 MHz, CDCl_3_ sol.) of the crudes obtained in the first 5 cycles of recycling.

As reported in Figure 1, after 6 h benzoic acid precipitated in the water and it was separated from the reaction mixture simply by filtration. The conversion is quantitative after the first three cycles (see Figure 2, spectra 1–3) and only in the 4th and 5th cycles a minimal residue of unreacted benzaldehyde (**1a**) was observed. (Figure 2, spectra 4 and 5). 

The purity of the benzoic acid was also checked by ICP-MS spectrometry, that evidenced the presence of traces of selenium (<0.07%) that were undetectable after one recrystallization. This evidence also explains the reduced efficiency of the catalytic system after 3 cycles. The protocol probably suffers of the combination of two factors: a small loss of catalyst and a progressive dilution due to the addition of new oxidant (30% hydrogen peroxide) at each cycle.

Worth summarizing in terms of the greenness of the proposed protocol is the fact that a gram scale synthesis and purification of benzoic acid starting from the corresponding aldehyde can be performed avoiding the use of organic solvents, using a stoichiometric amount of hydrogen peroxide, recycling and reusing at least five times the reaction medium containing the catalyst (overall yield of 87%).

## 3. Experimental Section 

### 3.1. General Information

Reactions were conducted in a round bottom flask and were stirred with Teflon-coated magnetic stirring bars at 800 rpm. Solvents and reagents were used as received unless otherwise noted. Analytical thin-layer chromatography (TLC) was performed on silica gel 60 F254 precoated aluminum foil sheets and visualized by UV irradiation or by KMnO_4_ staining. Silica gel Kieselgel 60 (70–230 mesh, Merck, Darmstadt, Germany) was used for column chromatography. NMR experiments were conducted at 25 °C with a Bruker DPX 200 spectrometer (Bruker, Milan, Italy) operating at 200 MHz for ^1^H, 50.31 MHz for ^13^C, and 94.07 MHz for ^19^F experiments or with a Bruker DRX spectrometer operating at 400 MHz for ^1^H, 100.62 MHz for ^13^C and 76 MHz for ^77^Se experiments. ^1^H and ^13^C chemical shifts (δ) are reported in parts per million (ppm), relative to TMS (δ = 0.0 ppm) and the residual solvent peak of CDCl_3_ (δ = 7.26 and 77.00 ppm in ^1^H and ^13^C-NMR, respectively). Data are reported as chemical shift (multiplicity, coupling constants where applicable, number of hydrogen atoms, and assignment where possible). Abbreviations are: s (singlet), d (doublet), t (triplet), q (quartet), dd (doublet of doublet), dt (doublet of triplet), tt (triplet of triplet), m (multiplet), br. s (broad signal). Coupling constants (*J*) are quoted in Hertz (Hz) to the nearest 0.1 Hz. Inductively coupled plasma mass spectrometry (ICP MS) analysis was performed with a Varian 700-ES series instrument inductively coupled plasma-optical emission spectrometer (Varian, Les Ulis Cedex, France). 

### 3.2. General Procedure for the Synthesis of Carboxylic Acids **2**

Diphenyl diselenide (**3**, 0.006 g; 0.02 mmol) was treated with H_2_O_2_ (30%·*w*/*w*, 0.1 mL, 1 mmol) and water (0.2 mL) and stirred at room temperature at 800 rpm until the discoloration of the reaction mixture; then, the aldehyde **1** (1 mmol) was added. After 6 h, the aqueous mixture was extracted three times with EtOAc (3 × 20 mL). The collected organic layers were dried over Na_2_SO_4_ and the solvent evaporated under reduced pressure.

*Benzoic acid* (**2a**). White solid, 99% yield, m.p. 122 °C [32], ^1^H-NMR (CDCl_3_) δ: 7.49 (dd, 2H, *J* = 7.6 and 6.8 Hz), 7.63 (t, 1H, *J* = 6.8 Hz), 8.13 (d, 2H, *J* = 7.6 Hz), 11.88 (brs, 1H); ^13^C-NMR (100 MHz, CDCl_3_) δ: 128.5, 129.3, 130.2, 133.8, 172.5.

*2-Hydroxybenzoic acid* (**2b**). White solid, 73% yield, m.p. 160 °C [33], ^1^H-NMR (DMSO-*d6*) δ: 6.96–6.90 (m, 2H), 7.53–7.49 (m, 1H), 7.80 (dd, *J* = 1.2, 5.7 Hz, 1H), 11.36 (brs, 1H), 13.87 (brs, 1H); ^13^C-NMR (DMSO-*d6*) δ: 113.5, 117.6, 119.7, 130.8, 136.1, 161.7, 172.4. 

*4-Methylbenzoic acid* (**2c**). Yellow solid, 85% yield, m.p. 179 °C [34], ^1^H-NMR (CDCl_3_) δ: 2.43 (s, 3H), 7.26 (d, 2H, *J* = 8.3 Hz), 7.99 (d, 2H, *J* = 8.3 Hz); ^13^C-NMR (CDCl_3_) δ: 21.8, 126.5, 129.1, 130.2, 144.5, 172.3.

*4-Methoxybenzoic acid* (**2d**). Colorless solid, 80% yield, m.p. 183 °C [35], ^1^H-NMR (DMSO-*d_6_*) δ: 3.82 (s, 3H), 7.00 (d, 2H, *J* = 8.8 Hz), 7.89 (d, 2H, *J* = 8.8 Hz), 12.61 (brs, 1H); ^13^C-NMR (DMSO-*d_6_*) δ: 55.4, 113.8, 123.0, 131.3, 162.8, 167.0.

*4-Nitrobenzoic acid* (**2e**). Colorless solid, 88% yield, m.p. 243 °C [36], ^1^H-NMR (DMSO-*d_6_*) δ: 8.10 (d, 2H, *J* = 8.3 Hz), 8.26 (d, 2H, *J* = 8.3 Hz), 13.61 (brs, 1H); ^13^C-NMR (DMSO-*d_6_*) δ 123.9, 130.9, 136.6, 150.2, 166.0.

*3-(Trifluoromethyl)benzoic acid* (**2f**). Colorless solid, 99% yield, m.p. 105 °C [37], ^1^H-NMR (DMSO-*d_6_*) δ: 7.76 (t, 1H, *J* = 8.0 Hz), 8.00 (d, 1H, *J* = 8.0 Hz), 8.17 (s, 1H), 8.22 (d, 1H, *J* = 8.0 Hz), 13.52 (br s, 1H); ^13^C-NMR (DMSO-*d_6_*) δ: 123.8 (q, *J*_C–F_ = 271 Hz), 125.5 (q, *J_C–_*_F_ = 4 Hz), 129.4 (q, *J*_C–F_ = 4 Hz), 129.5 (q, *J*_C–F_ = 32 Hz), 130.2, 131.9, 133.3, 166.1. ^19^F-NMR (DMSO-*d_6_*) 61.5 (s).

*3*,*5-Difluorobenzoic acid* (**2g**). Colorless solid, 78% yield, m.p. 121 °C [38], ^1^H-NMR (CDCl_3_) δ: 7.20 (tt, 1H, *J* = 3.0 and 9 Hz), 7.50–7.60 (m, 2H); ^13^C-NMR (CDCl_3_) δ: 109.4 (t, *J*_C–F_ = 25 Hz), 113.2 (m), 132.3 (t, *J*_C–F_ = 9 Hz), 162.8 (dd, *J*_C–F_ = 12 and 249 Hz), 170 (t, *J*_C–F_ = 3 Hz); ^19^F-NMR (DMSO-*d_6_*) 108 (s).

*2*,*2-Diphenylacetic acid* (**2h**) 80% yield, white solid, m.p. 143 °C [39], ^1^H-NMR (DMSO-*d_6_*) δ: 5.00 (s, 1H), 7.20–7.30 (m, 2H), 7.30–7.35 (m, 8H), 12.70 (br s, 1H), ^13^C-NMR (DMSO-*d_6_*) δ: 56.3, 126.8, 128.4, 128.5, 139.5, 173.4. 

*Cinnamic acid* (**2i**) obtained as *Z*/*E* mixture (76:24) in 85% yield. Major isomer (*Z*-**2i**) ^1^H-NMR (CDCl_3_) δ: 6.50 (d, 1H, *J* = 12.7), 7.25–7.35 (m, 3H), 7.50–7.55 (m, 2H), 7.85 (d, 1H, *J* = 12.7); ^13^C-NMR (CDCl_3_) δ: 118.7, 128.1, 129.4, 129.9, 134.4, 145.8, 171.3.

*(E)-Hex-2-enoic acid* (**2j**). Oil [40], 99% yield, ^1^H-NMR (CDCl3) δ: 0.95 (t, 3H, J = 7.4 Hz), 1.45–1.55 (m, 2H), 2.20–2.25 (m, 2H), 5.80 (d, 1H, *J* = 15.6 Hz), 7.05 (dt, 1H, *J* = 15.9 and 6.9 Hz,); ^13^C-NMR (CDCl_3_) δ: 13.8, 21.3, 34.4, 120.8, 152.4, 171.9.

*Octanoic acid* (**2k)**. Oil [41], 99% yield, ^1^H-NMR (CDCl_3_) δ: 0.90 (t, 3 H, *J* = 5.0 Hz), 1.30–1.35 (m, 8H), 1.60 (q, 2 H, *J* = 5.0 Hz), 2.35 (t, 2 H, J = 5.0 Hz), 11.10 (br s, 1H); ^13^C-NMR (CDCl_3_) δ: 14.1, 22.7, 24.8, 29.0, 29.1, 31.7, 34.2, 180.6.

*Hexenoic acid* (**2l**). Oil [42], 99% yield, ^1^H-NMR (CDCl_3_) δ: 0.91 (m, 3H), 1.30–1.35 (m, 4H), 1.65 (m, 2H), 2.35 (t, *J* = 7.5 Hz, 2H); ^13^C-NMR (CDCl_3_) δ: 24.4, 22.3, 13.8, 31.2, 34.0, 180.2

### 3.3. General Procedure for the Synthesis of Esters **6**

Diphenyl diselenide (**3**, 0.006 g; 0.02 mmol) was treated with H_2_O_2_ (30%·*w*/*w*, 0.15 mL, 1.5 mmol) and stirred at room temperature until the discoloration of the reaction mixture. Then, the aldehyde **1** (1 mmol) and the appropriate alcohol (2.5 mmol) were added. The reaction mixture was stirred at 50 °C for 2 h and extracted three times with EtOAc (3 × 20 mL). The collected organic layers were dried over Na_2_SO_4_ and the solvent evaporated under reduced pressure.

*Methyl benzoate* (**6a**). Colorless oil [31], 90% yield, ^1^H-NMR (CDCl_3_) δ: 3.88 (s, 3H,), 7.59–7.35 (m, 3H), 8.12–8.04 (m, 2H); ^13^C-NMR (CDCl_3_) δ: 52.4, 128.1, 129.3, 130.3, 132.6, 166.4.

*Methyl 4-methyl-1-benzoate* (**6c**). White solid, 75% yield, m.p. 34 °C [31], ^1^H-NMR (CDCl_3_) δ: 2.41(s, 3H), 3.90(s, 3H), 7.24(d, *J* = 8.0 Hz, 2H), 7.93(d, *J* = 8.0 Hz, 2H); ^13^C-NMR (CDCl_3_) δ: 21.5, 51.8, 127.4, 129.0, 129.5 143.4, 167.0.

*Methyl 4-methoxy-1-benzoate* (**6d**). Colorless oil [43], 10% yield, ^1^H-NMR (CDCl_3_) δ: 3.84 (s, 3H), 3.88 (s, 3H), 6.90 (d, 2H, *J* = 8.8 Hz), 7.98 (d, *J* = 8.8 Hz, 2H); ^13^C-NMR (CDCl_3_) δ: 51.8, 55.3, 113.5, 122.5, 131.5, 163.3, 166.9.

*Methyl 4-nitro-1-benzoate* (**6e**). White solid, 95% yield, m.p 90 °C [31], ^1^H-NMR (CDCl_3_) δ: 3.91 (s, 3H), 8.18 (d, *J* = 6.0 Hz, 2 H), 8.21 (d, *J* = 6.0 Hz, 2 H). ^13^C-NMR (CDCl_3_) δ: 52.8, 122.5, 130.7, 145.0, 150.5, 165.1.

*Methyl 3-(trifluoromethyl) benzoate* (**6f**). Colorless oil [44], 96% yield, ^1^H-NMR (CDCl_3_) δ: 3.95 (s, 3H), 7.55 (dd, *J* 7.8 and 7.8 Hz, 1H), 7.80 (d, *J* = 7.8 Hz, 1H), 8.22 (d, *J* = 7.8 Hz, 1H), 8.30 (s, 1H); ^13^C-NMR (CDCl_3_) δ: 52.7, 126.7, 129.3, 129.6, 131.2, 133.2, 133.6, 166.0.

*Ethyl benzoate* (**6g**). Colorless oil [45], 87% yield, ^1^H-NMR (CDCl_3_) δ: 1.38 (t, *J* = 7.2 Hz, 3H), 4.39 (q, *J* = 7.2 Hz, 2H), 7.40–7.45 (m, 2H), 7.55 (t, *J* = 7.4 Hz, 1H), 8.04 (d, *J* = 7.2 Hz, 2H); ^13^C-NMR (CDCl_3_) δ: 17.1, 60.3, 128.4, 130.3, 131.9, 133.6, 168.7.

*Propyl benzoate* (**6h**). Colorless oil [45], 80% yield, ^1^H-NMR (CDCl_3_) δ: 1.02 (t, *J* = 7.2 Hz, 3H), 1.75–1.80 (m, 2H), 4.25 (t, *J* = 6.8 Hz, 2H), 7.40–4.45 (m, 2H), 7.50–7.55 (m, 1H), 8.00–8.05 (m, 2H); ^13^C-NMR (CDCl_3_) δ: 10.5, 22.1, 66.5, 128.3, 129.5, 130.5, 132.8, 166.6.

*Isopropyl benzoate* (**6i**). Colorless oil [46], 77% yield, ^1^H-NMR (CDCl_3_) δ: 1.36 (s, 3H), 1.38 (s, 3H), 5.26 (m, 1H), 7.41–8.06 (m, 5H); ^13^C-NMR (CDCl_3_) δ: 22.2, 68.6, 128.5, 129.7, 131.1, 132.9, 166.3.

*Cyclohexyl benzoate* (**6j**). Colorless oil [47], 70% yield, ^1^H-NMR (CDCl_3_) δ: 1.35-2.00 (m, 10H), 5.00–5.05 (m, 1H), 7.40–7.55 (m, 3H), 8.10–8.15 (m, 2H); ^13^C-NMR (CDCl_3_) δ: 23.6, 25.4, 31.5, 72.9, 128.2, 129.4, 130.9, 132.6, 165.9.

*4-Methyl phenol* (**7c**). Yellowish solid,15% yield, m.p. 35 °C [48], ^1^H-NMR (CDCl_3_) δ: 2.27 (s, 3H). 5.56 (s, 1H), 6.74 (d, *J* = 8.4 Hz, 2H), 7.03 (d, *J* = 8.4 Hz, 2H); ^13^C-NMR (CDCl_3_) δ: 20.6, 115.3, 130.1, 130.3, 153.4.

*4-Metoxy phenol* (**7d**). Yellow solid, 35% yield, m.p. 55 °C [48], ^1^H-NMR (CDCl_3_) δ: 3.75 (s, 3H), 5.31 (s, 1H), 6.72–6.82 (m, 4H); ^13^C-NMR (CDCl_3_) δ: 56.0, 114.9, 116.1, 149.7, 153.5.

*4-Methoxyphenyl formate* (**8d**). It was obtained in 60% yield as a colorless solid after performing the reaction at 0 °C using an ice bath. m.p.: 30 °C [49], ^1^H-NMR (CDCl_3_) δ: 3.81 (s, 3H), 6.91 (d, *J* = 8.8 Hz, 2H), 7.05 (d, *J* = 8.8 Hz, 2H), 8.30 (s, 1H); ^13^C-NMR (CDCl_3_) δ: 55.5, 114.8, 121.5, 143.6, 156.4, 159.7.

## 4. Conclusions 

In conclusion, a new protocol was developed to prepare carboxylic acids directly from aldehydes selectively and in excellent yields using diluted hydrogen peroxide, diphenyl diselenide as the catalyst and water as the solvent. Even if the oxidation of aldehydes has been widely explored in the past, nowadays a number of very recent publications demonstrates that there is still an interest on this topic, mainly focused on the development of more sustainable and green protocols [50,51] as well as on the elucidation of the actual mechanism [52]. By changing the solvent for an alcohol instead water, it was possible to prepare esters in very good yields and high selectivity directly from aldehydes. The method is suitable for aromatic and aliphatic aldehydes and was successfully used in a gram scale preparation of benzoic acid from benzaldehyde in which the solvent/catalyst system was reused with good results for five successive reactions and no organic solvent was used in any step of the procedure, as benzoic acid was recovered from the reaction mixture just by simple filtration. It is reasonable to envision that this latter protocol can be used in all the cases in which the carboxylic acid can be directly crystalized in the reaction medium (e.g., starting from aromatic aldehydes) especially in the cases in which the oxidation proceeded in almost quantitative yields.
